# An Unfortunate Incident of 'Talk and Die': A Case Report

**DOI:** 10.7759/cureus.90047

**Published:** 2025-08-14

**Authors:** Utpal Tripura, Gambhir Singh, Arun Prakash

**Affiliations:** 1 Forensic Medicine, All India Institute of Medical Sciences, Kalyani, Kolkata, IND; 2 Forensic Medicine, SRM Medical College Hospital and Research Centre, Chennai, IND

**Keywords:** blunt injury, extradural hematoma, head injury, intracranial hemorrhage, lucid interval

## Abstract

Extradural hematoma (EDH) is an intracranial hemorrhage often caused by blunt trauma and may be associated with skull fractures. It is characterized by a "lucid interval," where patients briefly regain consciousness before sudden deterioration, a critical feature in forensic investigations. This case involves a 40-year-old woman who suffered blunt head trauma due to domestic abuse. After losing consciousness, she regained awareness and walked 2 km before being found deceased in a locked public toilet. Autopsy revealed a left temporo-parietal skull fracture and a large EDH, with death attributed to EDH secondary to blunt trauma. The victim's ability to walk during the lucid interval underscores the importance of timely diagnosis and 24-hour monitoring to prevent early discharge. This case highlights the medico-legal implications of volitional acts during the lucid interval, stressing the need for forensic awareness in crime reconstruction.

## Introduction

Head injury is a broad term encompassing a wide range of injuries affecting the scalp, skull, brain, underlying tissues, and blood vessels in the head, typically caused by mechanical forces. A blunt force impact to the head often results in scalp contusions, which usually involve the superficial fascia, temporalis muscles, or the loose areolar tissue between the galea aponeurotica and pericranium. Scalp contusions are generally more palpable than visible [1]. Intracranial hemorrhage refers to bleeding within the cranium. Epidural or extradural hemorrhage occurs above the dura mater but within the skull [2]. This condition is commonly associated with skull fractures, with the posterior branch of the middle meningeal artery being the most frequent source of bleeding [3]. Following the initial impact, there may be a brief loss of consciousness, followed by a lucid interval during which the individual appears to behave normally. However, as bleeding progresses, the condition can rapidly deteriorate and result in death, a phenomenon known as the ‘Talk and Die’ syndrome [4].

## Case presentation

A couple married for 20 years had frequent quarrels, and the husband, habitually returning home drunk, would often assault his wife. On the night in question, during an argument, the husband forcibly banged his wife's head against a wall, after which she collapsed on the floor unconscious, and the husband left home. This incident occurred around 8:30 p.m. Their two children attempted to wake her but were unsuccessful. They then went to inform their maternal grandparents, who lived 2 km away. When the children returned with their grandparents, the woman was no longer at home. Despite an extensive overnight search, she could not be located. The next morning, around 5:00 a.m., a missing person report was filed at the local police station. Later that same day, the police received another complaint regarding a nearby public toilet that had remained suspiciously locked from the inside for an extended period. Upon forcibly opening the door, they discovered a woman’s dead body lying in a corner of the toilet. Family members identified the body as that of the missing woman. A medico-legal autopsy of the deceased was conducted.

Autopsy findings

The deceased was identified as a Hindu female, about 40 years old. The body was covered in a white cloth and clothed in a saree, petticoat, and blouse. All clothes were intact and free from bloodstains. The body was of average build and nourishment. Postmortem staining was present on the dependent areas over the back and had become fixed. Rigor mortis was well-developed throughout the body. The eyes and mouth were closed. No external injuries were noted.

**Figure 1:**
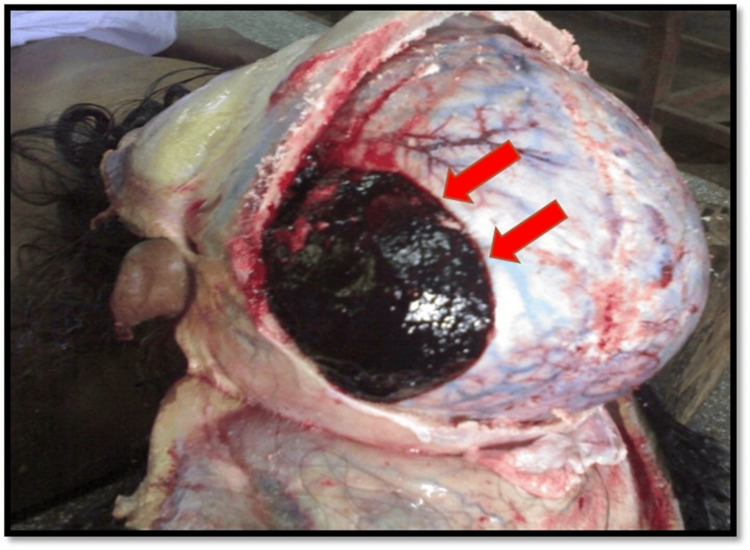
Extradural haemorrhage over left parieto-temporal region

Internal examination revealed a faint scalp contusion measuring 8 cm × 6 cm over the left parietal region. The underlying left temporo-parietal bone showed a linear antemortem fracture approximately 6 cm long. On opening the skull, an extradural hematoma measuring 15 cm × 14 cm with a maximum central thickness of 2.5 cm was found over the left temporo-parietal region (Figure 1). All brain meninges were intact. The brain appeared edematous and congested. There was no evidence of injury to the brain matter or any other intracerebral hemorrhage. All other internal organs appeared grossly normal. The cause of death was determined to be “death due to blunt force injury to the head.”

## Discussion

Extradural hematomas (EDH) occur in approximately 10.6% of traumatic brain injury cases admitted to hospitals [5,6], accounting for about 5-15% of all fatal head injuries [7]. They are more common in individuals under 50 years of age [8]. Most EDHs are associated with fractures of the underlying skull, although approximately 15% occur without skull fractures [8,9]. EDHs are frequently associated with a ‘lucid’ or ‘latent’ interval following the injury, during which symptoms may temporarily subside. In some cases, EDH may develop without any initial loss of consciousness. There may also be no lucid interval if unconsciousness due to compression immediately follows the concussion, without a phase of temporary recovery. The duration of the lucid interval is variable and may be absent in cases of prolonged concussion or concurrent brain damage [8].

Veevers et al. reported three cases of young adult males who died following blunt head and facial injuries. The victims managed to walk away from the scene of the assault before collapsing and dying nearby [10]. Unlike the present case, those victims had multiple visible head and facial injuries. Chacón-Aponte et al., in their review article, described the ‘Talk and Die’ syndrome in detail and noted that most victims were in the later half of life [11]. Reilly et al., in their study of “Patients with a head injury who talk and die,” found that 25% of 66 patients who were able to talk at some point after injury did not have an intracranial hematoma at autopsy [4]. These cases often showed localized swelling due to contusion. In about 50% of non-hematoma cases, ischemic or hypoxic brain damage was observed, typically without contusions [4,12,13].

Parkinson et al. reported four cases involving double lucid intervals associated with posterior fossa extradural hematomas [12]. Typically, deterioration from EDH either ends in death or requires surgical intervention [13-15]. However, in cases with double lucid intervals, the first phase of deterioration resolves spontaneously, resulting in a second brief lucid period, which is then followed by rapid decline of consciousness, as the hematoma expands or grows.

During the investigation, it was suspected to be a routine domestic quarrel, following which the woman might have left home out of fear and hidden inside the public toilet. The absence of sexual assault and any signs of struggle, along with the fact that the toilet was locked from the inside, ruled out the involvement of any third party. Given the children’s statements, autopsy findings, and crime scene evidence, it was concluded that the woman died from an extradural hematoma. It was deemed possible that during the lucid interval, she walked 2 km and locked herself inside the toilet, where she later died.

## Conclusions

Extradural hematoma creates challenging situations for both doctors and legal professionals because patients can seem fine after a head injury, only to suddenly worsen hours later. During this deceptive "lucid interval," victims may walk, talk, and act normally before collapsing unexpectedly. This can lead doctors to discharge patients too early or cause investigators to misunderstand what happened, especially in cases involving domestic violence. Every person with a head injury should be monitored in a hospital for at least 24 hours, no matter how minor it seems. Medical staff, police, and judges need a better understanding of how people can appear normal before deteriorating rapidly. Quick NCCT (non-contrast computed tomography) brain scans and careful observation can save lives and ensure justice is served properly.
